# Correction to: Serum heparan sulfate levels are elevated in endotoxemia

**DOI:** 10.1186/s40001-021-00600-z

**Published:** 2021-10-30

**Authors:** K. F. Hofmann-Kiefer, G. I. Kemming, D. Chappell, M. Flondor, H. Kisch-Wedel, A. Hanser, S. Pallivathukal, P. Conzen, M. Rehm

**Affiliations:** 1 Clinic of Anesthesiology/Critical Care Medicine and Pain therapy (M.A.B., P.C), Ludwig-Maximilians-University, Munich, Germany; 2 Department of Anesthesiology and Intensive Care Medicine, Kreiskliniken Günzburg-Krumbach, Günzburg, Germany; 3 Clinic of Pediatrics, Common Hospital, Augsburg, Germany; 4 Department of Neonatology, Clinic of Gynaecology and Obstetrics, Ludwig-Maximilians-University, Munich, Germany; 5 Institute for Surgical Research, Ludwig-Maximilians-University, Munich, Germany

## Correction to: Eur J Med Res (2009) 14: 526–531 https://doi.org/10.1186/2047-783X-14-12-526

Unfortunately, in the original publication of the article [1], the sixth author’s name was published with spell error. The corrected author name is given in the author group above.

The original article has been corrected.
